# Subtype-WGME enables whole-genome-wide multi-omics cancer subtyping

**DOI:** 10.1016/j.crmeth.2024.100781

**Published:** 2024-05-17

**Authors:** Hai Yang, Liang Zhao, Dongdong Li, Congcong An, Xiaoyang Fang, Yiwen Chen, Jingping Liu, Ting Xiao, Zhe Wang

**Affiliations:** 1 Department of Computer Science and Engineering, East China University of Science and Technology, Shanghai 200237, China; 2 Cornell Tech, Cornell University, New York, NY 14853, USA; 3 Center for Continuing and Lifelong Education, National University of Singapore, Singapore 119077, Singapore

**Keywords:** deep learning methods, molecular subtyping, whole-genome, multi-omics data integration, cancer biomarkers

## Abstract

We present an innovative strategy for integrating whole-genome-wide multi-omics data, which facilitates adaptive amalgamation by leveraging hidden layer features derived from high-dimensional omics data through a multi-task encoder. Empirical evaluations on eight benchmark cancer datasets substantiated that our proposed framework outstripped the comparative algorithms in cancer subtyping, delivering superior subtyping outcomes. Building upon these subtyping results, we establish a robust pipeline for identifying whole-genome-wide biomarkers, unearthing 195 significant biomarkers. Furthermore, we conduct an exhaustive analysis to assess the importance of each omic and non-coding region features at the whole-genome-wide level during cancer subtyping. Our investigation shows that both omics and non-coding region features substantially impact cancer development and survival prognosis. This study emphasizes the potential and practical implications of integrating genome-wide data in cancer research, demonstrating the potency of comprehensive genomic characterization. Additionally, our findings offer insightful perspectives for multi-omics analysis employing deep learning methodologies.

## Highlights

• Subtype-WGME utilizes whole-genome omics data for precise cancer subtyping
• Subtype-WGME integrates the MLP-Mixer to handle high-dimensional multi-omics data
• Subtype-WGME explores the role of various omics and non-coding regions
• Based on Subtype-WGME, we develop a robust biomarker discovery pipeline

## Motivation

Many investigations have aimed to harness multi-omics data to elucidate the molecular subtypes and pathogenesis of cancer. Several cancer subtyping models based on exome sequencing have emerged that strive to integrate diverse omics information. However, protein-coding genes represent approximately 2% of the entire human genome, and growing evidence highlights the pivotal role of non-coding regions in the context of complex diseases. We therefore propose an innovative multi-omics cancer subtyping deep network to overcome the limitations of traditional models that predominantly rely on exome data. This deep network is specifically designed to comprehensively characterize the full spectrum of genomic data at the whole-genome level, facilitating a more integrated understanding of cancer biology.

## Graphical abstract

**Figure:**
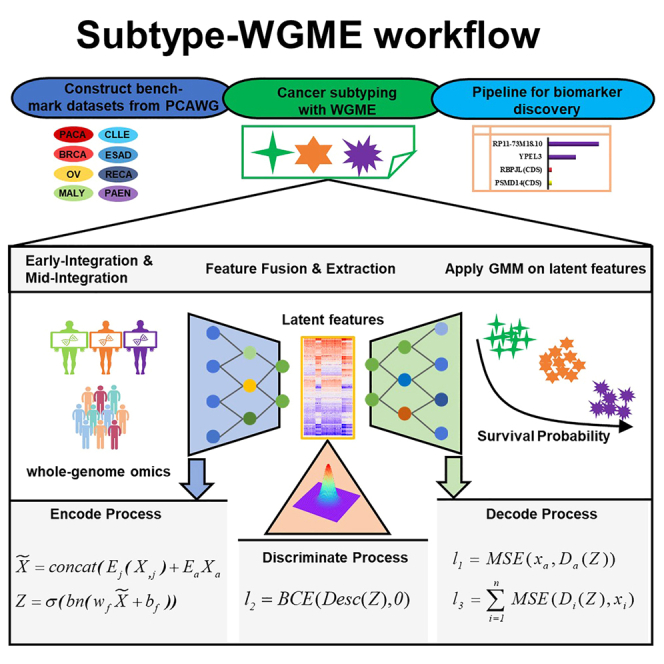

## Teaser

Yang et al. propose a cancer subtyping method using whole-genome multi-omics data, which extracts comprehensive features that capture the molecular subtypes of cancer. They also quantify the subtyping contribution of different omics and identify prognostic-related biomarkers. This approach expands the scope of cancer subtyping studies to the genome-wide level.

## Introduction

Cancer, a spectrum of complex genomic diseases, constitutes a formidable menace to human life and health.^1^ It is frequently characterized by gene mutations and concurrent molecular perturbations at the cellular level,^2^ such as alterations in gene and microRNA (miRNA) expression, as well as copy number variations. In the contemporary landscape of precision medicine, the application of targeted genomic therapies is indispensable for effective cancer treatment. However, a pronounced heterogeneity pervades both among patients with tumors and across different tumor types, and these variations can profoundly influence the clinical trajectories of patients. Cancer subtyping research endeavors to categorize cancers exhibiting similar phenotypes into distinct molecular subtypes, predicated on the molecular characteristics of tumor cells.^3^ These subtypes display identical biological properties and respond similarly to therapeutic interventions. The accurate delineation of molecular subtypes is of paramount importance in cancer diagnosis, prognosis, and the selection of appropriate treatments.^4^ Nevertheless, cancer subtyping investigations continue to present substantial challenges owing to the diversity, complexity, and specificity inherent in cancer genomics.

Cancer subtyping methods predicated on single-omics data neglect the shared attributes and disparities among patients’ multi-omics molecular profiles, thereby constraining the accuracy of the obtained results. In contrast, integrated subtyping approaches that incorporate multiple omics datasets capitalize on the complementary information derived from diverse molecular datasets to delineate cancer patients.^5^ In recent years, the rapid advancements in next-generation high-throughput sequencing technologies have propelled the field of cancer molecular subtyping. International cancer research endeavors, such as the International Cancer Genome Consortium (ICGC)^6^ and The Cancer Genome Atlas (TCGA),^7^ have played a pivotal role in advancing this field by providing extensive omics data and clinical information across various cancer types. These large-scale collaborative efforts have fostered the growth of cancer molecular subtyping research. Furthermore, recent studies have emphasized the importance of non-coding region data, which harbors valuable biological insights.^8^^,^^9^^,^^10^ Notably, The Pan-Cancer Analysis of Whole Genomes (PCAWG) project aggregated whole-genome sequencing data from 2,658 cancer cases spanning 38 tumor types. It constituted a collaborative endeavor between the ICGC and TCGA. The availability of genome-wide multi-omics data has expanded opportunities for cancer subtyping investigations.

**Figure 1:**
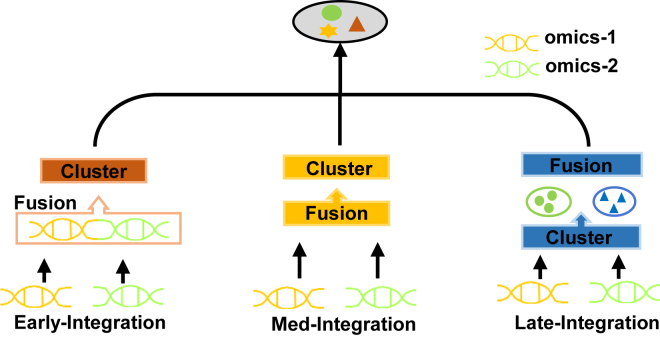
Three strategies for integrating multi-omics data in cancer subtyping

Several compelling subtyping methodologies have emerged, which can be classified into three distinct groups (Figure 1) based on the timing of integrating multi-omics data^11^: early integration (Early-Integration), medium integration (Med-Integration) and late integration (Late-Integration). Early-Integration commonly involves the elementary concatenation and amalgamation of multi-omics data, fusing the data into a unified input prior to modeling, followed by the application of clustering techniques such as K-means^12^ for classifying the integrated data. For instance, LRAcluster^13^ connects the heterogeneous multi-omics data for each sample by probabilistically modeling the distribution of numerical, count, and discrete features. However, Early-Integration tends to increase data dimensionality and overlooks the variability of different omics distributions.

Med-Integration approaches involve the internal fusion of multiple omics data types, aiming to establish a shared subspace across different omics data. MCCA^14^ and nonnegative matrix factorization (NMF)^15^ employ dimensionality reduction algorithms to condense multi-omics data into a shared low-dimensional subspace, to maximize the correlation between features, and subsequently to perform clustering. Based on deep neural networks, Subtype-GAN^16^ utilizes adversarial training to independently reduce the dimensionality of each omics datum and subsequently concatenate the low-dimensional representations for clustering. Subtype-WESLR^17^ integrates clustering knowledge from disparate methods using a weighted ensemble strategy, preserving the local structure of the original sample feature space. It ensures consistency with the weighted ensemble while mapping sample features from each omics to a common latent subspace. DLSF^18^ integrates multi-omics data by learning a coherent sample manifold space through a deep cycle autoencoder (CAE) framework with self-expression layers. These Med-Integration methods facilitate the exploration of shared patterns among different omics data types, enhancing the robustness and interpretability of cancer subtyping.

Late-Integration methods involve the independent clustering of different omics data types, followed by merging the results to generate a unified outcome. PINS,^19^ for instance, employs perturbation clustering to compress each omics data type and constructs a connectivity matrix for fusion. SNF^20^ computes and merges samples from each omic level based on similarity and conducts cluster analysis. NEMO^21^ introduces a neighborhood multi-omics clustering algorithm based on a similarity network, constructing a similarity matrix for each omic datum and computing the average similarity matrix across all data types. SUMO^22^ is a unique method that leverages NMF to cluster different omics data types. It addresses the challenge of missing omics data by employing multiple quality and stability metrics to determine consensus clustering labels. By integrating these metrics, SUMO provides an effective solution for accurately assigning cluster labels to samples, even in the absence of omics data. These Late-Integration approaches enable the integration of independent clustering results from different omics data, facilitating a comprehensive understanding of the complex interplay between various molecular attributes and their impact on cancer subtyping.

**Figure 2:**
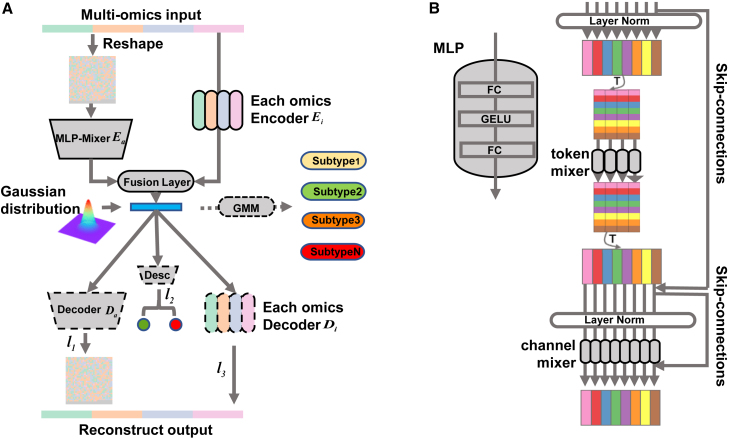
Summary of Subtype-WGME (A) Subtype-WGME is an unsupervised learning framework. The encoder comprises an MLP-Mixer and a multilayer perceptron (MLP). After training, clustering is performed using a Gaussian mixture model in the hidden space. (B) MLP-mixer model structure.

In current cancer subtyping research, the focus has primarily been on utilizing coding region data. In contrast, the potential role of non-coding region multi-omics data in cancer molecular subtyping remains largely unexplored. The analysis of whole-genome data poses challenges due to limited sample sizes and the high dimensionality of the data, making existing subtyping methods less suitable for such complex inputs. In this study, we aim to address these challenges and investigate the molecular subtyping of cancer using whole-genome multi-omics data, with a specific focus on understanding the contributions of non-coding data to cancer subtyping. To tackle the intricacies of whole-genome multi-omics data, we have developed an innovative deep learning model termed subtyping with the whole genome multi-omics encoder (Subtype-WGME) (STAR Methods; Figure 2). Subtype-WGME combines the MLP-Mixer^23^ network and the adversarial variational autoencoder^24^ structure to perform unsupervised dimensionality reduction of whole-genome multi-omics data. It learns a low-dimensional latent space that is consistent across different data modalities, enabling the exploration and interpretation of subtypes. Specifically, Subtype-WGME adeptly integrates high-dimensional multi-omics data using Early-Integration and Med-Integration strategies. It leverages the MLP-Mixer network, known for its capacity to handle complex multi-omics data representations in the latent space. We assembled a large sample size by collecting data from eight cancer types from the PCAWG dataset, creating eight benchmark datasets. The results demonstrate that Subtype-WGME surpasses the current state-of-the-art methods in cancer subtyping tasks. Furthermore, based on the subtyping outcomes, we developed a biomarker discovery pipeline leveraging the random forest algorithm. This pipeline allowed us to assess the importance of genome-wide data in the subtyping task and to identify biomarkers derived from whole-genome data. Our findings highlight the clinical relevance of non-coding region data and provide valuable insights for further research in the field of cancer subtyping.

## Results

### Performance on eight benchmark PCAWG cancer datasets

In our study, we conducted analyses on eight tumor datasets obtained from the PCAWG. The eight cancers are ovarian cancer (OV), pancreatic endocrine neoplasms (PAEN), renal cell cancer (RECA), chronic lymphocytic leukemia (CLLE), esophageal adenocarcinoma (ESAD), malignant lymphoma (MALY), pancreatic cancer (PACA), and breast cancer (BRCA), respectively, and their corresponding project codes are inside the parentheses. These datasets included four types of omics data: RNA sequencing data (RNA), single nucleotide mutation (Mut), copy number alteration (CNA), and miRNA expression (miRNA). Gene expression level obtained from RNA sequencing data reveals variations in gene transcript levels across samples, providing valuable insights for tumor subtype identification. We will refer to RNA sequencing data as gene expression throughout the following sections. Mutation data help identify genetic mutations associated with tumor development and enable the exploration of genetic differences between tumor subtypes. CNA data assist in revealing distinct patterns of gene copy number variations in tumors. Additionally, miRNA data allow for the identification of miRNA expression patterns associated with tumor subtypes, shedding light on the regulatory role of miRNA in tumorigenesis and tumor progression. We compared the performance of Subtype-WGME, our proposed method utilizing whole-genome multi-omics data, with four commonly used methods that rely on coding region data in multi-omics subtyping analyses. The comparative algorithms employed in the study consisted of Med-Integration methods such as Subtype-GAN and MCCA, as well as Late-Integration methods like NEMO and SNF. To ensure comparability across methodologies, we established an identical number of subtypes for each cancer type based on prior research, the detailed introduction of which can be found in STAR Methods. For further details on the dataset processing procedures, please refer to the STAR Methods and Tables S1 and S2.

**Figure 3:**
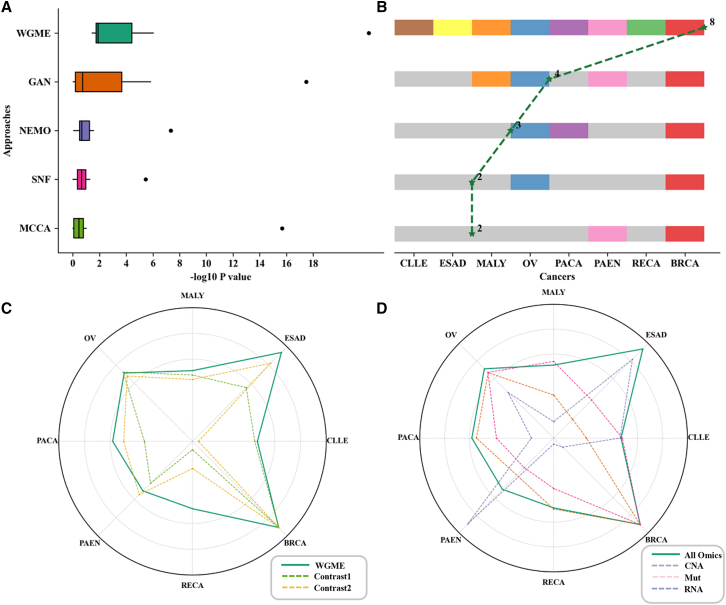
Performance results on the benchmark datasets (A) The comparative boxplots of survival analysis for the five methods. The x axis represents the −log10 *p* value, and the y axis represents the different methods. In this plot, WGME means Subtype-WGME, and GAN denotes Subtype-GAN. *p* value was determined via the log-rank test. (B) The dataset situation concerning significant subtyping results was procured by different methods. The y axis is the same as in (A). The gray color indicates that the dataset did not achieve significant survival analysis results, while the colored regions signify significant analysis results. (C) Performance comparison radar plots between Subtype-WGME and two ablation analyses. Log10 scaling is applied uniformly to *p* values for better visualization. (D) Performance comparison radar plots between using a single omic and using all four omics. Log10 scaling is applied uniformly to the *p* value for better visualization.

Firstly, we assessed the clustering results of different methods using −log10 *p* value (Figure 3A) and the number of cancer datasets with significant subtyping results (*p* value < 0.05) (Figure 3B). We conducted survival analysis on the subtyping results using the log rank test, an extension of the Cox proportional hazards model tailored for comparing the survival curves among multiple groups. The test statistic follows a chi-squared distribution, and the *p* value is computed accordingly. Subtype-WGME exhibited superior performance compared to all benchmark methods, achieving a higher median −log10 *p* value of 2.367 and an average of 7.06. It consistently demonstrated significant survival analysis results across all datasets. Specifically, Subtype-WGME yielded significant results for CLLE (3.01E−03), ESAD (6.24E−12), MALY (4.07E−02), OV (6.30E−11), PACA (4.55E−02), PAEN (2.27E−02), RECA (1.10E−02), and BRCA (2.19E−19). Subtype-GAN ranked second among all compared algorithms, with a median −log10 *p* value of 1.11 and an average of 3.49, and it displayed significant differences in survival analysis on half of the cancer datasets. NEMO ranked third, with a median −log10 *p* value of 0.811 and an average of 3.78, achieving significance in three datasets. SNF and MCCA followed. Overall, Subtype-WGME outperformed other methods regarding survival analysis performance. Additionally, we recorded the runtime of all methods. Due to the generally small sample sizes in the datasets, deep learning methods were slower than traditional models, consistent with previous findings in coding differentiation tasks.^25^ Subtype-WGME demonstrated superior performance with an average total running time of 25.36 s across eight cancer datasets, outperforming our previously proposed deep subtyping model, Subtype-GAN. When considering only the inference time, the average time further decreases to 2.16 s for detailed performance in survival analysis and runtime (Tables S3 and S4).

To assess the efficacy of Subtype-WGME in handling genome-wide high-dimensional data, we conducted two comparative analyses, where specific analysis data can be found in Table S5. In the first analysis, we eliminated the early fusion pipeline of Subtype-WGME, involving the encoding and decoding process using MLP-Mixer. Instead, we individually applied encoding and decoding to each single omic using a fully connected network. The results revealed that the median survival analysis of the subtyping outcomes was 1.43 when using only this single-stream network across eight datasets. This analysis marked a significant decrease compared to the median survival analysis of Subtype-WGME’s subtyping results, which was 2.367, underscoring the substantial contribution of the dual-stream network, incorporating both early and intermediate fusion, in enhancing subtyping performance. Illustrated in Figure 3C, relative to Subtype-WGME, comparison analysis 1 demonstrated notable variations in the survival analysis of subtyping outcomes for only four datasets, ESAD, MALY, OV, and BRCA, while exhibiting poorer results for the remaining seven datasets. In the second comparison analysis, we omitted the reshaping step for the concatenated multi-omics and directly employed a simple fully connected network for dimensionality reduction. This was done as a substitution for the MLP-Mixer network structure, allowing us to assess its significance within the model. The results indicate that the median survival analysis of the subtyping outcomes using the fully connected network on the eight datasets is 2.035, showcasing a noticeable performance decline. This underscores the rational and effective application of the MLP-Mixer network structure in our model. In particular, this analysis yielded notable distinctions in the survival analysis of subtyping outcomes for five datasets—ESAD, OV, PACA, PAEN, and BRCA—showcasing superior results on the remaining seven datasets, excluding PAEN, where Subtype-WGME exhibited less favorable subtyping outcomes.

In our final exploration of data subtyping from various omics, we conducted analyses using Subtype-WGME for each single omic and exploratory analyses using only coding and non-coding region data (Figure 3D; Table S5). The results suggest that optimal performance in cancer subtyping is achieved using RNA data alone, followed by Mut data and CNA data. Nevertheless, when employing all omics data for subtyping, the performance may not consistently surpass that of using single-omics data, as multi-omics data introduce increased noise. Nonetheless, Subtype-WGME adeptly harnesses the complementary information across multiple groups, leading to more robust and stable results. Specifically, the median survival analyses of the subtyping results for RNA, CNA, and Mut omics on the eight datasets were 1.725, 0.724, and 1.229, respectively. For the CLLE and MALY datasets, optimal subtyping results are attained when exclusively utilizing mutation data. In the case of the RECA dataset, the most accurate subtyping outcomes are achieved with the use of only RNA data. Conversely, for the PAEN dataset, the subtyping results demonstrate superior performance when exclusively relying on CNA data. Moreover, when considering only coding region data for subtyping, the result was 1.717, while subtyping with non-coding region data alone yielded a result of 0.839. Although the use of multi-omics coding region data significantly outperformed non-coding region data, there was an apparent decrease compared to using whole-genome data, underscoring the feasibility and necessity of whole-genome subtyping.

### Construction of the genome-wide cancer biomarker discovery pipeline

Expanding upon the insights derived from Subtype-WGME, we conducted further interpretative research on the subtype results using the random forest (RF) algorithm as described in STAR Methods (Figure S2). Moreover, we investigated the utilization of XGBoost and LightGBM as alternatives to the RF model (STAR Methods). We developed a pipeline for the automated exploration of whole-genome-wide biomarkers across various cancers. The process begins by splitting each dataset into a training dataset and a valid dataset with a proportion of 6:4. We utilize four omics features from the training set as input for the RF model, with subtype labels obtained from Subtype-WGME as the training output. The RF model is then trained using these data. Subsequently, we rank the omics data features based on the Gini importance score, selecting the top 50 features with the highest scores as an initial set of candidate biomarkers. For each candidate biomarker in the preliminary set, we segregate the samples into high- and low-expression groups based on the median expression level of the biomarker in the validation set. We calculate the relevance of the survival analysis by assessing the difference between these two groups and identifying features with significant results. These selected features constitute the final set of biomarkers. Finally, a comprehensive literature review is conducted on the final biomarkers to validate the credibility and rationale of the study further.

Our proposed pipeline identified a total of 195 biomarkers across all cancer datasets, and the statistics of biomarkers and the biomarkers found in each cancer are presented in Tables S9A and S9B-I. Among them, 157 biomarkers were located in coding regions, while 38 were located in non-coding regions. When categorized based on omics features, 144 biomarkers were associated with gene expression, 3 with copy number variation, and 45 with mutations. Notably, non-coding biomarkers were predominantly associated with long non-coding RNA (lncRNA), such as CTD-2192J16.26, RP11-134G8.8, SNORD116-20, and RP11-68I3.11. It has been observed that lncRNA plays a crucial role in regulating gene expression in coding regions^26^^,^^27^ and is also implicated in cancer subtyping.^28^ Additionally, we identified antisense genes with significant correlations to patients’ survival outcomes, such as RP5-894A10.2, RP11-380L11.4, MAP3K14-AS1, and others. Notably, RBFOX1 emerged as a biomarker applicable to both PAEN and MALY cancers. Furthermore, we categorized Mut and CNA omics features into more specific regions, including coding DNA sequence (CDS), promoter core region (PromCore), 5′ untranslated region (5′ UTR), 3′ untranslated region (3′ UTR), enhancers, splice site (SS), and non-coding RNA (ncRNA) (STAR Methods). Our analysis revealed that mutations in both coding and non-coding regions can serve as effective biomarkers for cancer subtyping. For instance, mutations in the PromCore and CDS of CNTNAP2, as well as mutations in the 5′ UTR and CDS of RBFOX1, can function as biomarkers for PAEN. Similarly, mutations in the 5′ UTR and CDS region of GRID2 and mutations in the 3′ UTR and CDS of USH2A can serve as biomarkers for MALY.

In a conclusive phase, we meticulously validated the potential biomarkers identified by our pipeline through an extensive literature review, confirming the credibility of 20 among them (Table 1; Table S9J). For esophageal cancer patients, the downregulation of ABI3BP^29^ was observed to inhibit cancer cell proliferation, activity, migration, and invasion, classifying it as an oncogene pivotal in the progression of growth and metastasis. In the context of MALY, PTPRD,^30^^,^^31^ a recognized tumor suppressor gene, exerted regulatory control over cell growth, with its mutations being proposed as promising diagnostic biomarkers for MALY. In the ovarian cancer domain, potential biomarkers included ZNF217,^32^ implicated in cancer cell proliferation, invasion, and metastasis, and RBM, an upregulated gene acting as a regulator of variable pre-mRNA splicing, thereby influencing apoptosis through the modulation of apoptotic factors. For PACA patients, the identified biomarkers comprised MMP28,^33^ contributing significantly to the tumor microenvironment, CYP3A5,^34^ serving as a predictor of treatment response, TP53BP1,^35^ which exhibited inhibitory effects on pancreatic tumor growth, and ROBO2,^36^ a regulator of TGF-β in the pancreas. Additionally, CNTNAP2,^37^ with recurrent mutations, emerged as a potential prognostic biomarker, while PDE4D^38^ was identified as an independent prognostic factor, and GPC5^39^ correlated significantly with favorable survival outcomes. Renal cancer patients featured biomarkers such as RPL36A^40^ and TACC3,^41^ with associations with immune-related pathways and T cell depletion. Within the realm of BRCA patients, GNPNAT1^42^ surfaced as an upregulated biomarker associated with proliferation and invasiveness, CD22^43^ exhibited heightened expression, correlating with tumor size, DHCR24^44^ overexpression was linked to tumor growth promotion, and BUB3^45^ was upregulated and negatively correlated with various immune cell types. Furthermore, low PHF2^46^ expression was identified as significantly associated with poor prognosis, while FAM83H-AS1^47^ and lncRNA-ATB were observed to be overexpressed in sera, demonstrating correlations with tumor metastasis, size, and lymph node metastasis and emphasizing their prognostic rather than diagnostic value.

**Table 1:** Biomarkers after literature validation

Biomarker	Gini score	*p* value	Comment
ABI3BP	1.52E−02	2.50E−03	ESAD, CNA, SS
PTPRD	8.74E−03	2.16E−02	MALY, Mut, 5′ UTR
ZNF217	7.16E−03	1.18E−03	OV, RNA
RBM25	7.07E−03	8.99E−05	OV, RNA
MMP28	5.68E−03	2.74E−02	PACA, RNA
CYP3A5	5.09E−03	2.74E−02	PACA, RNA
TP53BP1	4.81E−03	2.74E−02	PACA, RNA
ROBO2	1.33E−02	4.33E−02	PAEN, Mut, SS
ROBO2	1.10E−02	4.33E−02	PAEN, Mut, CDS
CNTNAP2	5.66E−03	1.73E−02	PAEN, Mut, 5′ UTR
PDE4D	5.16E−03	3.33E−03	PAEN, Mut, SS
GPC5	3.54E−03	1.72E−02	PAEN, Mut, SS
CNTNAP2	4.77E−03	3.72E−02	PAEN, Mut, CDS
RPL36A	1.02E−02	2.51E−02	RECA, RNA
TACC3	8.15E−03	2.67E−02	RECA, RNA
GNPNAT1	1.34E−02	1.09E−02	BRCA, RNA
CD22	7.87E−03	1.49E−02	BRCA, RNA
DHCR24	7.70E−03	3.63E−03	BRCA, RNA
BUB3	6.96E−03	7.82E−03	BRCA, RNA
PHF2	6.96E−03	1.09E−02	BRCA, RNA
FAM83H-AS1	6.56E−03	7.82E−03	BRCA, RNA

### Importance analysis of the four omics

To investigate the influence of different omics data on tumor subtyping results at the genome-wide level, we summarized and quantified the Gini importance scores of each omics datum, thereby determining their contributions to the final subtyping outcomes. Due to the absence of miRNA data in the six datasets and their limited contribution to the subtyping of individual cancers (with the highest percentage not exceeding 2% and an overall percentage not exceeding 1%), our study primarily focused on analyzing the contributions of RNA, Mut, and CNA omics data to different cancer subtyping.

**Figure 4:**
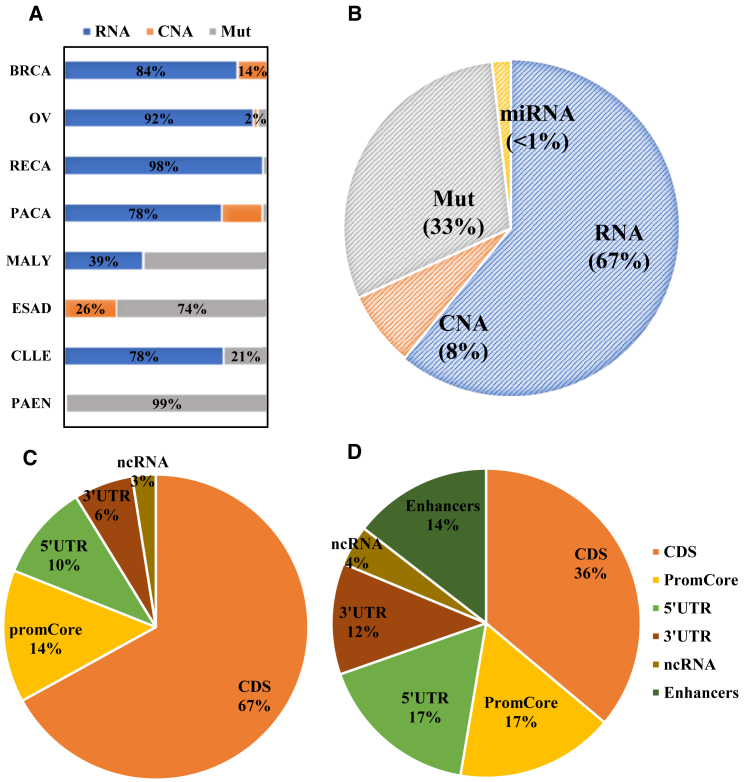
Analysis of omics importance in cancer subtyping (A) Importance of RNA, Mut, and CNA in the eight cancers. (B) The overall contribution of RNA, Mut, and CNA in all eight cancers. (C) Importance of mutation in the pan-cancer datasets. (D) Importance of CNA omic in pan-cancer datasets.

Our analysis revealed that genome-wide multi-omics data’s relative contributions in different cancer datasets were correlated with cancer types (Figure 4A). Considering all cancers (Figure 4B), gene expression data accounted for 58% of the total. They played the most significant role in the six datasets, specifically in BRCA (84%), OV (92%), RECA (98%), PACA (78%), MALY (39%), and CLLE (78%). This result suggests that gene expression levels dominate tumor subtyping, consistent with previous findings regarding coding regions.^48^^,^^49^ It’s noteworthy that RNA omics contribute less than 1% in both the ESAD and PAEN datasets. This is primarily due to the prevalence of missing data in the original RNA data. After preprocessing, all available PAEN samples exhibit missing values for RNA data, with only a handful of ESAD samples retaining RNA data. Notably, attempts to subtype the ESAD dataset using RNA omics alone failed to achieve statistical significance. Despite substantial data gaps in the original dataset, our model adeptly extracts complementary information from multi-omics sources for classification. This underscores the compatibility of Subtype-WGME with challenges posed by missing data issues. Furthermore, mutation data contributed 33% in combination in the context of genome-wide cancer subtyping. In comparison, CNAs contributed 8%, indicating the greater importance of mutation data than CNAs (Table S6).

Moreover, the contribution of different genomic regions to the subtyping results was analyzed to assess the importance of non-coding region features that were previously overlooked. Specifically, we examined the contribution of mutations and CNAs in the above genomic regions (Table S7). Our findings revealed consistent patterns of contribution across different cancer datasets. Considering all datasets together, the CNAs in the CDS region were crucial, accounting for 36% of the contribution (Figure 4D). In three individual datasets (PACA, ESAD, and PAEN), the CNAs in the CDS region were relatively more important than in other loci. In terms of mutation data, mutations in the CDS region were also pivotal (Figure 4C), contributing to 67% among all the datasets, and in all eight datasets, mutations in the CDS region were relatively more important compared to other loci. Additionally, we observed that variations in the gene promoter and 5′ UTR also played significant roles in subtyping. In mutation data, the contribution of the promoter interval was 14%, ranking second, while the 5′ UTR contributed 10% and ranked third. The subsequent rankings were 3′ UTR (6%) and ncRNA (3%). Similarly, in copy number omics, the promoter interval accounted for 17% of the contribution, ranking second, followed by 5′ UTR (17%) in the third place. The subsequent rankings were enhancers (17%), 3′ UTR (12%), and ncRNA (4%). Promoter activity holds paramount significance in cancer subtyping as it governs the timing and expression levels of genes by interacting with transcription factors, thus dictating gene activity. Promoters have been demonstrated to play pivotal pathological roles in diverse cancers, such as BRCA^50^ and bladder cancer.^51^ Additionally, the 5′ UTR plays a pivotal role in modulating gene expression levels, and previous studies indicate that the DNA sequence of the 5′ UTR encompasses numerous *cis*-regulatory elements that engage with transcription factors. The spectrum of mutations in the 5′ UTR collectively influences multiple mechanisms of gene expression, holding significant functional implications in cancer.^52^

### Case study on OV

**Figure 5:**
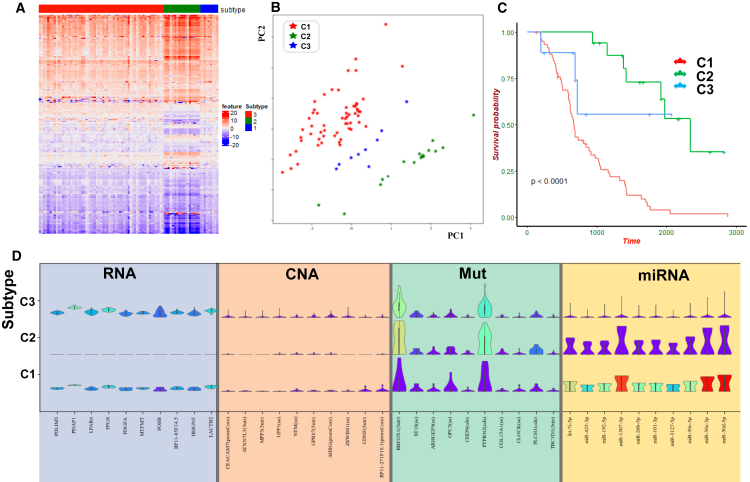
Interpretable analysis on OV cancer (A) Heatmap depicting the differences in cryptic features and subtype labels. (B) Using the PCA algorithm to visualize the hidden layer features after reducing dimensionality. (C) Survival curves of the three subtypes of OV cancer, which was plotted by Kaplan-Meier estimate analysis. (D) Violin plots showing the distribution of selected biomarkers’ features in the three subtypes.

We conducted a detailed analysis of the Subtype-WGME subtyping results on ovarian cancer to demonstrate the rationality of the classification. Firstly, we plotted a heatmap of differences between the latent feature representations and subtypes to observe the degree of aggregation between subtypes (Figure 5A). It was evident that there were distinct boundaries between the low-dimensional features of patients from different subtypes, indicating that different subtypes were well-reflected in the latent space. To further visualize the latent features of the three subtypes, we used the PCA algorithm to project the latent vectors from 256 dimensions to 2 dimensions. We envisioned the 2-dimensional features and sample subtype labels (Figure 5B). It could be observed that samples belonging to the same subtype were clustered together. In contrast, samples from different subtypes were widely separated: the visual representation of the latent features provided an intuitive distinction between subtypes.

Moreover, we performed Kaplan-Meier survival analysis on the OV cancer dataset to demonstrate significant differences in survival times among samples corresponding to different subtypes (Figure 5C). The three subtypes exhibited distinct survival patterns, with a substantial difference (*p* value = 9.6E−07). Specifically, Subtype-WGME classified OV tumors into three subtypes, denoted as subtypes C1, C2, and C3, and sample labels are listed in Table S8. Subtype-C1 comprised 59 patients, of which 54 had died, with an average survival time of 804 days; subtype-C2 has 17 patients, with only deaths and an average survival time of 1,731 days; subtype-C3 included nine patients, 3 of whom have died, with an average survival time of 871 days. These results indicate that Subtype-WGME can effectively distinguish OV tumor subtypes without prior knowledge (such as the number of subtypes and patient age). Through the above visualizations, we provided compelling evidence that the latent features extracted by Subtype-WGME contain biologically relevant information correlating with subtypes. The identified subtypes are meaningful and interpretable.

Additionally, we utilized the Gini score to identify the top 10 features in each omics dataset and illustrated the distribution of the three subtypes based on their original expression (Figure 5D). The results showed significant differences in the distribution of these genomic features among the three subtypes, suggesting a correlation between genomic features and subtypes. Moreover, different genomic features exhibited distinct distribution patterns among the three subtypes, complementing each other and collectively contributing to the final subtyping results.

**Figure 6:**
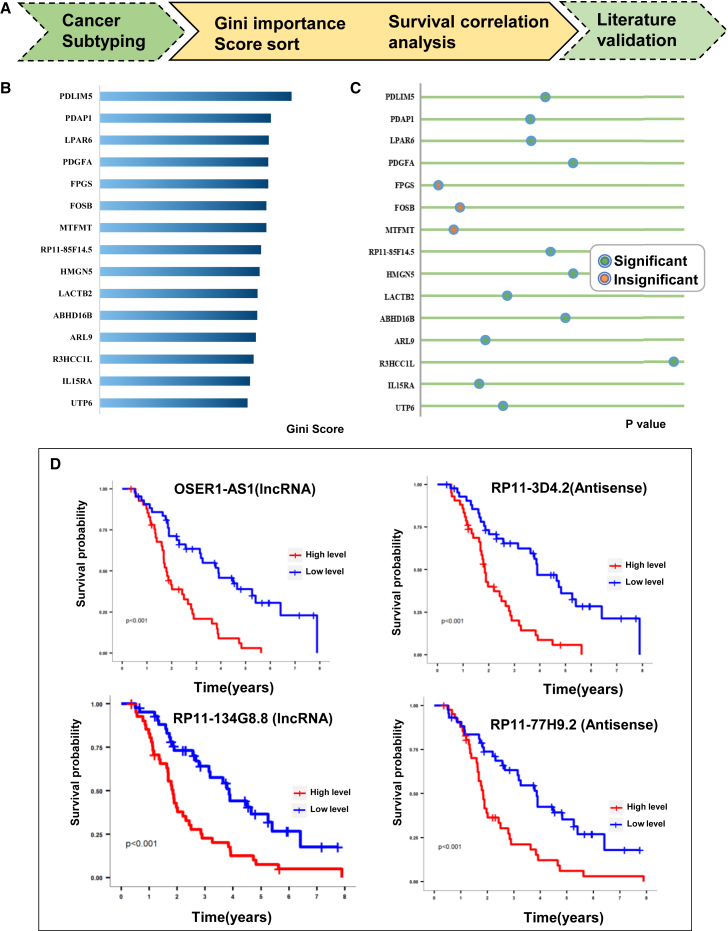
Biomarker analysis on OV cancer (A) The pipeline for genome-wide biomarker discovery in the OV dataset. (B) The top important features obtained after training the random forest algorithm. The brackets indicate the occurrence of mutations or copy number variants in the corresponding interval. (C) The significance of survival differences between high and low expression groups based on feature values. Aqua green color indicates significance, while the orange color indicates insignificance. (D) The biomarker discovery pipeline identified four non-coding region biomarkers for OV cancer.

Based on the subtyping results, we applied the proposed biomarker pipeline to the OV dataset (Figure 6A). Initially, we selected a preliminary set of 50 biomarkers based on their Gini importance ranking on the training set (Figure 6B). Subsequently, we divided the samples into two subgroups based on the expression levels of these biomarkers and performed survival analysis to validate their relevance on the validation set. Among the 50 selected biomarkers, 31 exhibited significant differences in survival analysis (Figure 6C), indicating their potential as prognostic indicators. Further analysis of these 31 biomarkers revealed eight potential non-coding biomarkers with clinically relevant survival curves (Figure 6D). To elaborate, three LncRNAs, OSER1-AS1 (1.85E−03), RP11-134G8.8 (9.2E−05), and CTD-2192J16.26 (4.95E−02), as well as four antisense genes, RP11-85F14.5 (6.53E−03), RP11-3D4.2 (2.96E−02), RP11-77H9.2 (4.49E−02), and RP5-894A10.2 (1.28E−02), and one pseudogene, RP11-91I11.1 (2.12E−02), have been identified with significant relevance in the context of ovarian cancer subtyping. These findings underscore the intricate involvement of non-coding elements, such as LncRNAs, antisense genes, and pseudogenes, in the molecular landscape of ovarian cancer, highlighting their potential as crucial biomarkers in discerning distinct subtypes.

## Discussion

One of the paramount objectives in cancer genomics research is the integration of multi-omics data for the analysis of cancer subtypes and the identification of cancer biomarkers. With the rapid advancements in high-throughput sequencing technologies, we can now acquire comprehensive genome-wide multi-omics data, including RNA sequencing data, CNAs, and somatic mutations. Integrating these diverse yet complementary datasets allows for a more profound understanding of cancer progression and metastasis mechanisms. This study introduces an innovative whole-genome cancer subtyping method named Subtype-WGME, which is based on genome-wide data. This method combines multi-omics data to extract essential biological information from the vast high-dimensional multi-omics data and to capture higher-level complex associations within low-level features. To the best of our knowledge, Subtype-WGME is the first method that utilizes genome-wide multi-omics data for cancer molecular subtyping. By employing this approach, we have achieved optimal performance on eight cancer datasets from the PCAWG project. Moreover, our method demonstrated significance across all eight datasets. We also successfully identified 90 biomarkers that significantly impact patient survival time. These findings highlight the ability of Subtype-WGME to accurately characterize large-scale genome-wide multi-omics data and its enormous potential in discovering potential cancer targets on a genome-wide scale.

Subtype-WGME introduces an innovative deep learning architecture capable of integrating multiple inputs. In contrast to previous methods, it leverages both Early-Integration and Med-Integration data as inputs, employing different encoders tailored to each input type. This unique design allows the model to harness the strengths of both input modes, leading to improved performance. Particularly for high-dimensional multi-omics data, Subtype-WGME innovatively transforms the data into matrix form and applies MLP-Mixer for feature modeling. This approach reduces complexity and facilitates the modeling of omics information across multiple omics layers. Furthermore, Subtype-WGME incorporates regularization techniques into its output. Employing a discriminator with variational capabilities maps the latent features from diverse omics data, characterized by different distributions, into a unified space. The model robustly extracts multi-scale biological information from genome-wide multi-omics data throughout the training process. The resulting low-dimensional features adhere to a Gaussian distribution, enabling a unified feature representation that dramatically enhances the performance of subsequent clustering tasks.

We propose a biomarker discovery pipeline based on the RF algorithm in response to the subtyping results. This pipeline integrates genome-wide feature analysis and deep learning models to systematically explore features at various levels in the cancer genome, intending to identify potential cancer biomarkers. By considering both coding and non-coding region features, we gain a more comprehensive understanding of the complexity and diversity of cancer, thereby revealing previously undiscovered biological mechanisms. The pipeline enables the high-throughput discovery of potential cancer biomarkers. In addition to coding region features, we have identified a substantial number of cancer biomarkers in the non-coding regions, including antisense genes and lncRNA. Besides, we found that the coding and non-coding region features of CNTNAP2 and RBFOX1 can be used as biomarkers simultaneously. This finding challenges the conventional notion of exclusively focusing on protein-coding regions in biomarker studies. It offers a more profound understanding of the mechanisms and functional implications of gene regulation in cancer development. Unveiling these refined biomarkers constitutes a groundbreaking discovery that has yet to be explored in existing research, promising to propel the field of cancer precision medicine forward. These findings demonstrate the feasibility of employing deep learning models in genome-wide biomarker discovery. Further validation of these biomarkers will contribute to the realization of gene-specific therapies for cancer patients and propel the development of precision medicine, opening up exciting prospects for improved patient outcomes.

Furthermore, we quantitatively assessed the contributions of the four types of omics data involved in the analysis to cancer subtyping. The task of subtype delineation was primarily influenced by RNA sequencing data, followed by single nucleotide mutation data and CNA. However, the contribution of miRNA features was relatively low, which could be due to the large number of missing miRNA features in the data published by PCAWG. Gene expression data provide crucial information about gene activity within cancer cells and play a vital role in unraveling tumors’ molecular characteristics and dysregulations. Even at the genome-wide level, RNA sequencing data maintain their decisive role in the process of cancer subtyping. Although copy number data exhibit relative abundance, they are susceptible to substantial noise. On the other hand, gene mutations possess higher specificity, rendering them more influential than copy number variations. Additionally, a more granular analysis of somatic mutations and copy number variations revealed that changes occurring in the coding regions were paramount in determining the subtyping results. Such coding region variations directly impact gene mutations and structural alterations, influencing gene function and regulatory mechanisms. Simultaneously, the role of promoters in subtyping results proved to be of utmost significance. Promoters constitute crucial sequence regions that regulate gene transcription, controlling the initiation timing and extent of gene expression through interactions with transcription factors. It is important to note that while the significance of non-coding regions received preliminary validation in our study, further study is required to delve deeper into their specific mechanisms and functions.

### Limitations of the study

Although Subtype-WGME has achieved significant accomplishments in genome-wide data analysis, it also has certain limitations. Firstly, the presence of missing data can restrict the performance of the subtyping method, even to the extent of impeding its proper functioning. Currently, Subtype-WGME addresses missing data through a simple imputation method without considering the interrelationships among multiple omics data. The presence of missing data can potentially impact the accuracy of the model. This limitation will be addressed by leveraging the rapidly evolving AI-generated content model, which aims to complement missing data at the multi-omics level. Secondly, due to the nature of deep learning models, Subtype-WGME requires adequate training data to achieve optimal cancer subtyping performance. Insufficient training samples may adversely affect the model’s final performance. However, the availability of comprehensive genome-wide multi-omics data currently needs to be improved. Therefore, future study efforts will focus on enhancing the performance of Subtype-WGME through data augmentation techniques that leverage multi-omics integration. These considerations highlight areas for improvement and further exploration to overcome the limitations of Subtype-WGME and to enhance its effectiveness in cancer subtyping.

## STAR★Methods

### Key resources table

**Table:** 

REAGENT or RESOURCE	SOURCE	IDENTIFIER
**Deposited data**		

Gene expression, somatic mutation, miRNA expression, copy number alteration	ICGC Data Portal	https://dcc.icgc.org/releases/PCAWG/

**Software and algorithms**		

R v4.2.3	The R Foundation	https://www.r-project.org/
Subtype-WGME	This paper	https://zenodo.org/doi/10.5281/zenodo.11044332
NEMO	Rappoport et al.^21^	https://github.com/Shamir-Lab/NEMO
Subtype-GAN	Yang et al.^16^	https://github.com/haiyang1986/Subtype-GAN
MCCA from PMA package v1.2.1	Lee et al.^14^	https://cran.r-project.org/web/packages/PMA
SNF v2.3.1	Wang et al.^20^	https://cran.r-project.org/src/contrib/Archive/SNFtool/

**Other**		

Code to reproduce comparison of various methods	This Paper	https://github.com/zhaol233/Subtype-WGME/tree/master/other
Capsule website for reproducing Subtype-WGME	Code ocean	https://codeocean.com/capsule/4686472/tree/

### Resource availability

#### Lead contact

Further information and requests for resources should be directed to and will be fulfilled by the lead contact, Zhe Wang (wangzhe@ecust.edu.cn).

#### Materials availability

This study did not generate new unique reagents.

#### Data and code availability

• This paper analyzes existing, publicly available datasets processed and hosted on ICGC data portal https://dcc.icgc.org/releases/PCAWG/. Information is also listed in the key resources table.
• All original code has been deposited at GitHub and is publicly available as of the date of publication. Idedntifiers are listed in the key resources table.
• Any additional information required to reanalyze the data reported in this paper is available from the lead contact upon request.

### Method details

#### Method overview

Subtype-WGME leverages comprehensive genome-wide multi-omics data, including gene expression (RNA), miRNA, copy number (CNA), and mutation data (Mut) obtained from PCAWG cancer samples. To enhance the modeling capacity of sample features in the context of genome-wide multi-omics data, we have developed a multi-task auto-encoder framework with MLP-Mixer serving as the core encoder. This framework facilitates the fusion of reduced-dimensional holistic and single-omics features within a unified hidden Gaussian space, effectively capturing both the global associations and local variations of the samples. Consequently, the downstream tasks exhibit superior performance when dealing with high-dimensional and heterogeneous biological data. The resulting low-dimensional representations serve as feature representations for the sample’s multi-omics data, which are subsequently clustered using a Gaussian mixture model. A graphical representation of the Subtype-WGME model can be found in Figure 2.

#### Architecture of Subtype-WGME

The Subtype-WGME framework comprises three essential processes: encoding (E), discriminating (D), and decoding (D). In the encoding process, the primary objective is to reduce the dimensionality of input features and try to retain biological information, encompassing a pipeline that integrates multi-omics input(MLP-Mixer) and a pipeline for each omics data (Each-omics). For the integration of multi-omics input, the output is initially reshaped into a two-dimensional matrix, followed by encoding through the innovative MLP-Mixer Module for enhanced adaptability to high-dimensional data. In Each-omics pipeline, different dimensions of features are aligned using a specific fully connected layer, reducing them to the corresponding latent space. The encoding process also encompasses a fusion layer facilitating the merging of the output from both pipelines. This involves concatenating the outputs of the two pipelines, utilizing the MLP block for dimensionality reduction, and ultimately ensuring a consistent distribution of hidden layer features through Batch Normalization. This process aims to facilitate more effective integration of information. The GELU activation function (σ) is also utilized to enhance the fusion layer’s nonlinear representation capability. Let’s denote the *j* th omics data of the *i* th sample as $X_{ij}$. $X_{:j}$ represents the feature of the *j* th omics, $X_{i}$ represents the feature of the *j* th sample, and $X_{a}$ denotes the overall input obtained after combining multi-omics. Thus, the encoding process can be mathematically expressed as follows:

$$bn\left(x_{i}\right)=\frac{x_{i}-E\left[x_{i}\right]}{\sqrt{Var\left[x_{i}\right]}+\varepsilon }\cdot \gamma +\beta$$

$$\sigma =0.5\cdot \left(1+\tanh\left[\sqrt{\frac{2}{\pi }}\left(x+0.044715x^{3}\right)\right]\right)$$

$$Z=\sigma \cdot bn\left(w_{f}\left(concat\left(E_{j}\left(X_{j}\right)+E_{a}\left(X_{a}\right)\right)+b_{f}\right)\right)$$

Where $w_{f}$ and $b_{f}$ represent the parameters of the fusion layer, $E_{j}$ and $E_{a}$ illustrates the encoder for each omics and multi-omics features, *Z* denotes the extracted hidden layer features, concat denotes stitching together the implicit representation of each omics.

In the discriminating process, we apply variational processing to the hidden layer features to ensure that the encoder’s output follows a specific probability distribution in the low-dimensional confidential space. We select the standard normal distribution N(0,1) as the target distribution since any data distribution can be seen as a combination of multiple normal distributions. The discriminator assesses whether the hidden features conform to the normal distribution by determining if they belong to the distribution or not. Instead of using the Kullback-Leibler (KL) divergence, we employ adversarial training with binary cross-entropy (BCE) as the loss function.

In the decoding process, we reconstruct the hidden layer features to restore the original input. Similar to the encoding process, the decoding stage comprises two pipelines. These pipelines individually reconstruct the hidden layer features into the original omics data and the reshaped two-dimensional matrix resulting from the combined omics. Through this multi-tasking approach, our goal is to enhance the model’s learning capabilities. The overall loss function of the model consists of three components, as described as follows:

$$MSE=\frac{1}{n}\sum_{i=1}^{m}w_{i}{\left(y_{i}-{\hat{y}}_{i}\right)}^{2}$$

$$BCE=-\left(1-y\right)\log\left(1-\hat{y}\right)-y\;\log\hat{y}$$

$$l_{1}=MSE\left(X_{a},D_{a}\left(Z\right)\right)$$

$$l_{2}=KL\left(p_{\theta }\left(Z|X\right)\|\eta \left(0,1\right)\right)∼BCE\left(Desc\left(Z\right),0\right)$$

$$l_{3}=\sum_{j=1}^{n}MSE\left(D_{j}\left(Z\right),{\hat{X}}_{j}\right)$$

$$L=\lambda_{1}l_{1}+\lambda_{2}l_{2}+\lambda_{3}l_{3}$$

Where $D_{i}$ denotes each-omics decoder, $D_{a}$ denotes multi-omics decoder, $l_{1}$ denotes reconstruction loss of MLP-Mixer pipeline, $l_{2}$ denotes loss of discriminator, $l_{3}$ denotes reconstruction loss of each omics pipeline, $\lambda_{1}$, $\lambda_{2}$, $\lambda_{3}$ are hyperparameters, denotes the weight of each loss function.

#### MLP-Mixer Module

To address the challenges of high-dimensional multi-omics input, where fully connected layers alone may not effectively extract the underlying nonlinear biological association information and involve high computational complexity, we propose an innovative approach to reshape the multi-omics data into two-dimensional matrix, which can be seen as a image, for input representation. To reshape the data into a image with regular size (H,W), we pad the overall data with 1 to achieve the desired size. Subsequently, we partition the image into patches of equal measure, denoted as *p*, and reshape them into a matrix with dimensions $X_{a}\in R^{B\times N\times p^{2}}$, *B* represents the batch size. We employ a convolutional network to embed the matrix during the encoding process. MLP-Mixer utilizes the resulting sequence of patches with uniform embeddings as input. Each sample yields a two-dimensional representation:

$$H=W=\lceil \sqrt{\frac{D}{p}}\rceil *p$$

$$N=\frac{H*W}{p^{2}}$$

Where *D* represents the sum of the dimensions of all omics, *N* indicates the number of patches per sample.

The MLP-Mixer architecture is structured with several mixer layers of equal size, each composed of two pivotal components. The token mixer, where token denotes the smaller matrix in the image, strategically operates on the columns of the input tensor $X_{a}$. Its primary objective is to facilitate inter-token communication by adeptly fusing spatial information. Notably, the parameters of this process are shared across all columns to ensure model parameter efficiency. Concurrently, the channel mixer operates on the rows of the input tensor $X_{a}$, fostering information exchange between different channels, with shared parameters across all rows. Each mixer process comprises two fully connected layers. Following the initial fully connected layer, LayerNorm is applied to normalize features, enhancing model stability. Subsequently, the second fully connected layer undergoes processing, incorporating the nonlinear activation function GELU to introduce the model’s nonlinear transformation capability. This meticulous design permits seamless information flow and diverse nonlinear transformations within MLP-Mixer. Collectively, the architecture of MLP-Mixer is meticulously crafted to efficiently model and learn from input data. The encoding process of the MLP-Mixer can be summarized as follows:

$$U_{*,i}=Z_{*,i}+W_{2}\sigma \left(W_{1}LayerNorm{\left(X\right)}_{*,i}\right),\ldots i=1\ldots C\text{,}$$

$$Y_{j,*}=U_{j,*}+W_{4}\sigma \left(W_{3}LayerNorm{\left(U\right)}_{j,*}\right),\ldots j=1\ldots N\text{.}$$

$$Z_{a}=mean\left(Y_{*,i}\right),\ldots fori=1\ldots C\text{.}$$

Where $W_{1}$, $W_{2}$ denotes the parameters of the two fully connected layers of the MLP block in the token mixer process, $W_{3}$, $W_{4}$ denotes the parameters of the two fully connected layers of the MLP block in the channel mixer process.

#### Gaussian mixture model

Subtype-WGME utilizes the extracted feature representations to analyze complex omics data. In this study, the Gaussian mixture model (GMM) is employed for clustering. GMM maximizes the parameters (i.e., variance and mean) of the likelihood function. GMM refers to a linear combination of multiple Gaussian distribution functions. It has the ability to fit various types of distributions and is commonly used for scenarios where data within the same dataset exhibit different distributions or variations in the same distribution. Let X be a random variable, and the GMM can be represented by following equation:

$$p\left(x\right)=\sum_{k=1}^{K}\pi_{k}N\left(x|\mu_{k},\Sigma_{k}\right)$$

Where each component is denoted by $\left(x|\mu_{k},\Sigma_{k}\right)$ , $\mu_{k}$ is the mixture coefficient.

During the clustering process, GMM assumes that the spatial probability distribution of input features conforms to a mixed Gaussian distribution. The k components in the GMM correspond to the *k* clusters, making it a soft clustering method. The number of clusters needs to be specified. The parameter estimation uses the Expectation Maximization (EM) algorithm to determine the values of $\pi_{k}$, μ, and $\Sigma_{k}$. Subsequently, the GMM model assigns a probability to each sample belonging to each cluster. After the training process, the most suitable subtype labels are assigned based on the highest probability density of each sample across different clusters.

#### Benchmark method

The scripts for comparing various methods on this benchmark are available at https://github.com/zhaol233/Subtype-WGME. Subtype-GAN was directly implemented based on our previous work. NEMO, SNF, and MCCA are implemented in R. We conducted the experiments using R version 4.2.3. For the NEMO method, we downloaded the source code of the method from https://github.com/ShamirLab/NEMO and executed the NEMO method using the nemo.clustering function. For the SNF method, we used the SNFtool package (version 2.3.1), and for the MCCA method, we utilized the PMA package (version 1.2.1) and executed the PMA::MultiCCA function at runtime. The parameter settings for the comparison algorithms followed the recommended default configurations.

#### Random forest

A random forest is a collection of decision trees, where each decision tree is built using a subset of features randomly selected from the training set. Each tree is trained independently and makes predictions based on a different subset of features. The training process of a random forest is depicted in Figure S2. The random forest algorithm has three primary hyperparameters: node size, number of trees, and the number of features sampled. This study implemented the random forest algorithm using the scikit-learn library, with n_estimators set to 100 and max_depth set to 10. In the realm of random forests, a node marks a pivotal juncture in a decision tree, encapsulating a specific condition within the dataset. By leveraging a distinct feature and its corresponding threshold, each node meticulously partitions the dataset into two subsets. This iterative process unfolds, birthing additional child nodes, until a predefined stopping criterion is met. On the flip side, a leaf stands as the conclusive terminus of the decision tree, abstaining from further divisions. Each leaf node crystallizes into a definitive classification or regression outcome within the dataset. To gauge the importance of each feature, we delve into the realm of calculating the mean shift in node impurity across all decision trees. Impurity mirrors the extent to which distinct classes permeate the dataset, commonly gauged using metrics such as the Gini index or information gain. Throughout the feature importance computation, our focal point is discerning the cumulative impact each feature wields on node impurity across multiple decision trees via the split operation. This evaluation method accurately measures the contribution of each feature to the classification of subtypes. Suppose there are m features, and the Gini index for each feature is calculated using the following formula:

$$GI_{m}=1-\sum_{k=1}^{\left|K\right|}p_{mk}^{2}$$

Where *K* represents the number of classification categories, and $P_{m,k}$ represents the proportion of category *k* in node *m*.

#### Determining the number of cancer subtypes

In determining the number of cancer subtypes, we adhered to a standardized criterion by drawing insights from prior studies. Specifically, Subtype-WELSR^53^ utilized the silhouette index to ascertain the optimal number of ovarian cancer subtypes as 3 through clustering. Bloehdorn^54^ employed ensemble clustering on CLL 89 GEP data, achieving optimal subtype differentiation at k = 6. Liu,^55^ through proteomic analysis, classified esophageal cancer into two molecular subtypes: S1 and S2. Notably, the S2 subtype, characterized by the upregulation of spliceosomal and ribosomal proteins, exhibits a more aggressive nature. Loeffler-Wirth,^56^ referencing the 5th edition of the WHO classification of haemato-lymphoid tumors in 2022, categorized lymphoma into three main subtypes: BL, DLBCL, and FL. Zhao^57^ analyzed the whole transcriptome data of over 1,200 pancreatic cancer patients, identifying six subtypes through the nonnegative matrix factorization (NMF) clustering method to explore molecular heterogeneity. DLSF^18^ utilized spectral clustering on hidden layer features and eigengap analysis to delineate four subtypes of kidney cancer. Lehmann^58^ employed the TNBCtype tool, categorizing TCGA breast cancer data into four categories based on centroid correlation.

#### Biomarker discovery method analysis

The choice of the random forest algorithm is grounded in our specific context—dealing with high-dimensional data and a limited sample size. Random forests demonstrate excellence in handling smaller datasets with a large number of features. The ensemble nature of the algorithm mitigates the risk of overfitting, especially in scenarios with limited samples, by aggregating predictions from multiple subtrees and avoiding undue reliance on individual instances. This algorithm is widely applied in biomedical data analysis,^59^^,^^60^ aiding in the evaluation of feature importance. While continuing to explore alternative algorithms, such as XGBoost^61^ and LightGBM,^62^ for biomarker discovery, we conducted a specific comparison of the preselected biomarkers identified by these three algorithms. This comparison involved assessing the number of top 50 features, scored by each algorithm, that exhibited significant survival analysis results on the validation set (Table S10). Across 8 datasets, the random forest algorithm identified a total of 195 significant features, XGBoost 117, LightGBM 139. Notably, the random forest algorithm outperformed the other two algorithms in terms of the number of significant features found in 6 datasets. This highlights the robust capabilities of the random forest algorithm in our specific scenario.

### Quantification and statistical analysis

#### Data preprocessing

The dataset used in this study was obtained from PCAWG (https://dcc.icgc.org/pcawg). However, due to missing data, some cancer samples lacked clinical information, rendering it impossible to validate the model’s classification accuracy. The sample screening process is depicted in Figure S1A. After eliminating samples with missing clinical data, the study focused on eight cancer datasets with larger sample sizes: 91 BRCA tumors, 85 OV tumors, 83 PAEN tumors, 89 RECA tumors, 94 CLLE tumors, 98 ESAD tumors, 101 MALY tumors, and 235 PACA tumors. For the complete number of available samples for PCAWG cancers, please refer to Table S1. The dimension of genome-wide omics data is immense compared to the sample size, with 121,311 dimensions for Mutations (Mut), 1,864 dimensions for miRNA, 134,074 dimensions for Copy Number Alterations (CNA), and 112,171 dimensions for RNA data (Figure S1D). Moreover, the data distribution is highly imbalanced (Figure S1C). During the feature selection process (Figure S1B), we initially applied a logarithmic transformation to the RNA and miRNA data. Since the corresponding omics data were partially missing in most samples, we filled the missing data with zeros. Subsequently, we performed variance filtering (with a threshold of 0.2) to select the omics features for the eight cancer types of interest. This filtering step effectively reduces the input’s dimensionality (Table S2). Lastly, we applied *Z* score normalization to standardize the features by subtracting their mean and scaling them to unit variance.

#### Explanation of CNA and Mut regions

While constructing the biomarker identification pipeline, we used a dataset consistent with the subtyping task. For copy number alterations (CNA) and somatic mutation features, the PCAWG dataset provided locus information for each feature (Figure S3). The features included Coding DNA Sequence (CDS), Promoter Core Region (PromCore), 5′ untranslated region (5′UTR), 3′ untranslated region (3′UTR), Enhancers, non-coding RNA (ncRNA), and splice site (SS).

Gene structure primarily comprises two main regions: the coding and non-coding regions. The general DNA sequence from the transcription start site to the transcription termination site is called the protein-coding region sequence (CDS). In eukaryotes, the coding region is discontinuous and consists of exons and introns. Exons represent the DNA segments retained after preRNA undergoes splicing or modifications, and they ultimately appear in the mature RNA gene sequence. The non-coding region is vital in gene expression regulation and encompasses various functional elements such as promoters, enhancers, and UTR. Promoters are specific DNA regions that initiate transcription and are typically located upstream of the gene’s transcription start site. During transcription, RNA polymerase and transcription factors recognize and bind to specific DNA sequences within the promoter region, thus initiating transcription. On the other hand, enhancers are DNA sequences typically located at the transcription start site or within a 1 Mbp range downstream of the gene. Transcriptional activators can bind them. By binding to enhancers, these transcription factors increase the probability of gene transcription. Enhancers are found extensively in the gene structures of both prokaryotes and eukaryotes. UTR is also part of the non-coding region and can be transcribed without being translated into proteins. UTR is present at the coding region’s 5′ and 3′ ends. The 5′UTR refers to the sequence located upstream of the coding region, while the 3′UTR refers to the downstream sequence. UTR plays important roles in post-transcriptional regulation, including RNA stability, localization, and translation efficiency. In predicting biomarkers, enhancer biomarkers were not considered in this study because the features in the Enhancer region could not be directly mapped to corresponding genes. Additionally, when calculating the contribution of different loci to subtyping, the Splice Site feature, located within the CDS region, was categorized within the CDS intervals for comparison.

#### Experimental environment

During the training process of Subtype-WGME, we employed the backpropagation algorithm to optimize the parameters of the entire model iteratively. The objective was to minimize the losses of the discriminator and decoder, with the loss function hyperparameters set to 1, 1, and 0.01, respectively. We utilized the Adam optimizer with a learning rate of 0.005 for optimization. The training was performed with a batch size of 64. To determine the optimal training rounds, we implemented the early stop algorithm. Upon completing the training process, we obtained each sample’s low-dimensional hidden feature representations, which are crucial for subsequent analysis.

In Subtype-WGME, the Scikit-learn package version 0.24.2 was employed for implementing the Gaussian Mixture clustering method. The PyTorch package version was 1.9.0, the Timm version was 0.5.4, the Pandas version was 1.2.4, and the Numpy version was 1.24.2. The analysis was conducted using Google Colab, with the operating system being Ubuntu 20.04 Linux. The CPU used was Intel(R) Xeon(R) CPU @ 2.30GHz, and the GPU utilized was the NVIDIA Tesla T4.

## Supplemental information

Document S1. Figures S1–S3 and Tables S1–S8 and S10

Table S9. Biomarker information found by Subtype-WGME for each cancer, related to Table 1

Document S2. Article plus supplemental information
