# Supplementary material for: Subtype-WGME enables whole-genome-wide multi-omics cancer subtyping
Source: Cell Rep Methods. 2024 May 17;4(6):100781. doi: 10.1016/j.crmeth.2024.100781 (PMC11228280; doi:10.1016/j.crmeth.2024.100781)
Supplement: Document S1. Figures S1–S3 and Tables S1–S8 and S10 [file mmc1.pdf]

Cell Reports Methods, Volume *4*

Supplemental information

Subtype-WGME enables whole-genome-wide multi-omics cancer subtyping

Hai Yang, Liang Zhao, Dongdong Li, Congcong An, Xiaoyang Fang, Yiwen Chen, Jingping Liu, Ting Xiao, and Zhe Wang

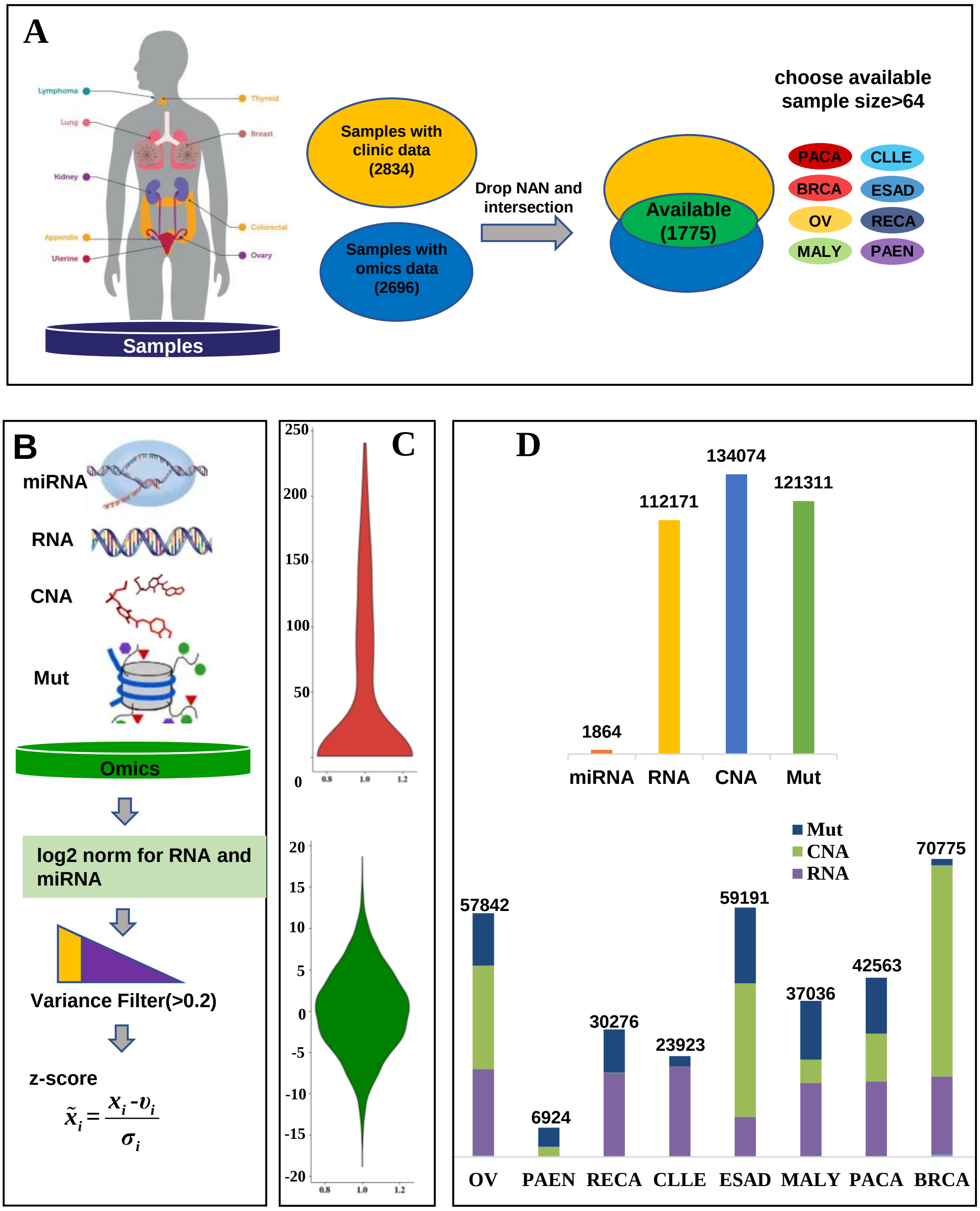

Figure S1. Data Set Construction Process, Related to data processing in STAR Methods. (A) Sample Selection Process. From the initial set of 2834 samples, we selected the samples with both clinical and omics data. We took the intersection of these two sets to obtain the final set of samples for analysis. (B) Feature Processing Process. (C) Distribution of features Before and After Using Z-score. The Z-score normalization process involved calculating each feature's mean and standard deviation and then transforming the values based on the Z-score formula. (D) The dimensionality of features before and after variance filtering.

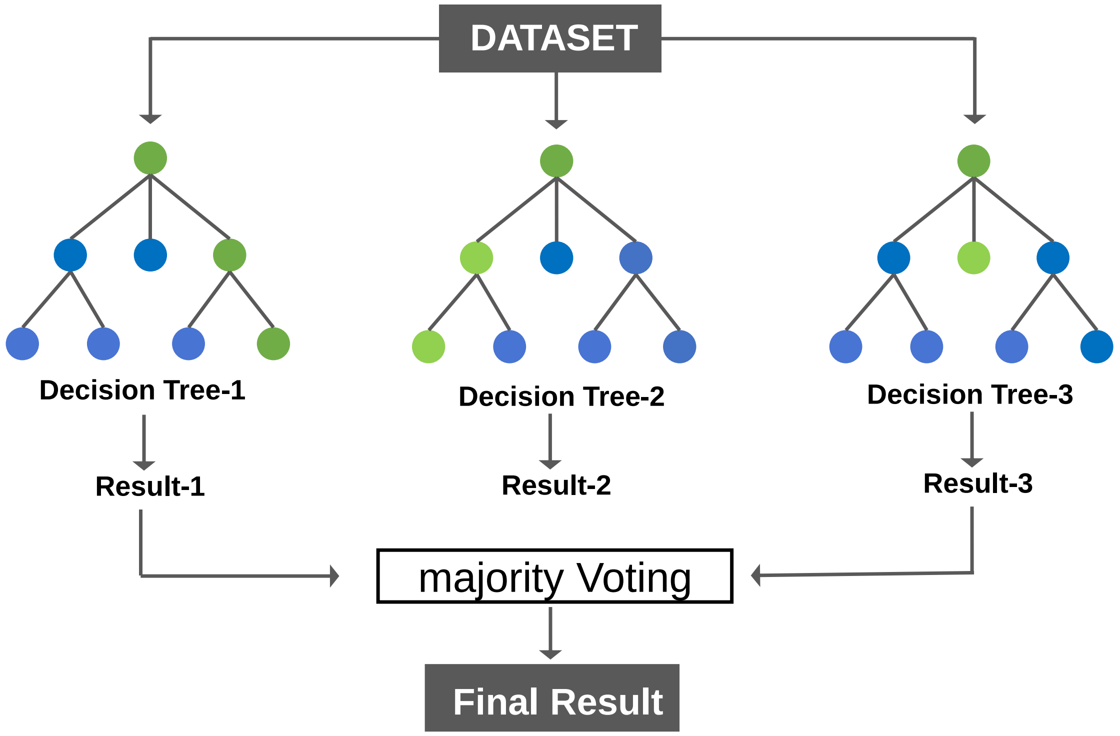

Figure S2. Inference process of the random forest algorithm, Related to random forest algorithm in STAR Methods. A random forest consists of multiple decision trees. Each tree is built independently using a random subset of the training data and a random subset of the features. The random forest algorithm combines the predictions from each tree through voting. Feature importance is calculated by averaging the contribution of each feature across all the trees in the forest.

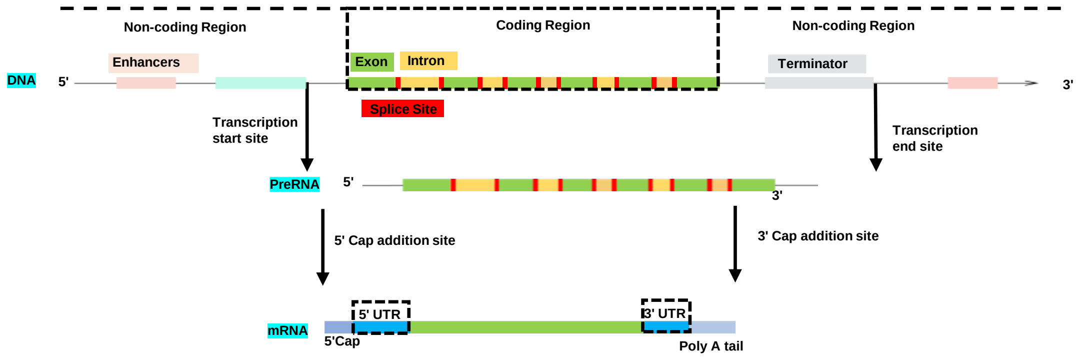

Figure S3. Information on loci involved in CNA and Mut omics data, Related to the explanation of CNA and Mut regions in the STAR Methods.

Table S1. All cancer total sample numbers and available sample numbers, Related to and STAR Methods.

**Code**	**Total Samples**	**Avaliable Samples**
LIRI	257	0
PBCA	250	42
**PACA**	**244**	**238**
**BRCA**	**217**	**91**
PRAD	177	53
**OV**	**118**	**86**
**MALY**	**101**	**101**
**CLLE**	**100**	**97**
**ESAD**	**100**	**100**
**RECA**	**95**	**95**
**PAEN**	**86**	**84**
BOCA	76	0
MELA	70	69
LIHC	54	39
UCEC	51	41
THCA	50	47
KICH	49	45
LUSC	48	31
COAD	46	40
HNSC	44	33
EOPC	44	39
LAML	43	27
GACA	42	41
LUAD	42	25
GBM	41	10
KIRC	40	29
STAD	39	36
SKCM	38	16
KIRP	34	32
SARC	34	25
CMDI	32	32
LINC	31	31
Null	25	0
BLCA	23	14
CESC	20	14
LGG	19	12
READ	16	16
ORCA	13	13
BTCA	12	0
DLBC	7	7

LICA	6	6

Table S2. Details of the PCAWG datasets used in the benchmark, Related to Figure S1A and STAR Methods.

Cancer	Name	Samples	miRNA expression	Gene expression	Copy number alteration	SNV/indel mutation
OV	Ovarian cancer	85	291	20566	24572	12413
PAEN	Rare Pancreatic Tumors	84	0	0	2407	4517
RECA	Renal cancer	89	0	19955	42	10279
CLLE	Chronic Lymphocytic Leukemia	94	0	21463	22	2438
ESAD	Esophageal cancer	98	0	9514	31673	18004
MALY	Malignant Lymphoma	101	0	17565	5524	13947
PACA	Pancreatic cancer	235	0	17943	11331	13289
BRCA	Breast Cancer	91	526	18595	50103	1551

Table S3. Survival analysis results from the benchmark datasets for the various methods, Related to Figure 3A and Figure 3B.

Code	Subtype-WGME	Subtype-GAN	NEMO	SNF	MCCA
CLLE	3.47E-02	4.52E-01	2.15E-01	4.83E-01	1.00E-01
ESAD	6.93E-23	9.18E-01	3.85E-01	2.08E-01	9.55E-01
MALY	1.27E-02	1.00E-05	2.49E-01	2.21E-01	9.29E-01
OV	9.57E-07	1.48E-06	4.61E-08	3.56E-06	3.49E-01
PACA	1.45E-03	1.85E-01	2.81E-02	4.38E-01	2.75E-01
PAEN	1.43E-02	3.33E-18	1.11E-01	5.46E-02	2.15E-16
RECA	2.33E-02	9.06E-01	8.61E-01	9.66E-01	8.17E-01
BRCA	2.19E-19	1.02E-02	2.19E-19	2.19E-19	6.79E-05

Table S4. Benchmark method running time, seconds, Related to Figure 3.

Code	Subtype-WGME	Subtype-GAN	NEMO	SNF	MCCA
CLLE	38.48	59.88	0.39	0.44	12.05
ESAD	22.94	68.04	0.76	0.86	6.52
MALY	27.60	62.10	0.58	0.68	5.98

OV	23.46	68.84	0.53	0.61	9.35
PACA	33.98	63.30	2.24	3.22	29.81
PAEN	13.89	46.46	0.07	0.10	0.66
RECA	15.87	57.24	0.29	0.33	6.25
BRCA	26.63	71.91	0.85	0.95	18.42
mean	25.36	62.22	0.71	0.90	11.13

Table S5. Survival analysis results from the contrast experiment, Related to Figure 3C and Figure 3D.

Code	Subtype-WGME	Contrast1	Contrast2	RNA	Mut	CNA	coding	noncoding
CLLE	3.47E-02	5.00E-02	7.85E-01	5.08E-01	2.82E-02	4.01E-02	2.00E-01	4.82E-01
ESAD	6.93E-23	4.02E-03	6.24E-12	6.00E-01	1.68E-01	6.24E-12	7.43E-02	3.88E-01
MALY	1.27E-02	2.76E-02	5.27E-02	3.37E-01	5.71E-03	7.28E-01	1.77E-01	2.14E-01
OV	9.57E-07	3.89E-07	1.08E-05	2.13E-05	1.75E-05	5.40E-02	2.46E-05	6.61E-03
PACA	1.45E-03	2.05E-01	1.82E-02	5.08E-03	1.23E-01	6.61E-01	2.10E-03	9.53E-02
PAEN	1.43E-02	7.33E-02	4.69E-03	-	3.60E-01	1.47E-18	4.95E-03	4.38E-01
RECA	2.33E-02	7.64E-01	5.36E-01	1.88E-02	2.18E-01	8.22E-01	7.28E-01	2.72E-02
BRCA	2.19E-19	2.19E-19	2.19E-19	2.19E-19	2.19E-19	7.66E-01	2.19E-19	9.82E-02

Table S6. Omics importance among eight cancers during Subtype-WGME, Related to Figure 4A and Figure 4B.

Code	miRNA	RNA	CNA	Mut
PAEN	-	-	1%	99%
CLLE	-	78%	<1%	21%
ESAD	-	<1%	26%	74%
MALY	-	39%	<1%	61%
PACA	-	78%	20%	2%
RECA	-	98%	<1%	2%
OV	1%	92%	2%	5%
BRCA	2%	84%	14%	<1%
Total	3%	469%	63%	264%
Proportion	0.3%	58.6%	7.9%	33.2%

Table S7. Region importance for copy number alteration and mutation, Related to Figure 4C and Figure 4D.

**CNA**						
**Code**	CDS	PromCore	5'UTR	3'UTR	ncRNA	Enhancers
PAEN	0.004085	0.003539	0.000995	0	0.000693	0.00144
CLLE	0	0.000547	0	0	0	0
ESAD	0.121212	0.030303	0.045455	0.015152	0.015152	0.030303
MALY	0.000582	0.000526	0.000575	0	0	0.001137
PACA	0.067985	0.028346	0.034539	0.037875	0.006137	0.024071
RECA	0	0	0	0	0	0
OV	0.004763	0.005434	0.003304	0.003558	0	0.002385
BRCA	0.030301	0.03624	0.022776	0.01726	0.004726	0.032622
**Mut**						
**Code**	CDS	PromCore	5'UTR	3'UTR	ncRNA	Enhancers
PAEN	0.639423877	0.175717341	0.094833	0.055092352	0.024182116	0
CLLE	0.139557	0.034352669	0.021948	0.010960667	0.008055016	0
ESAD	0.545455	0.075758	0.045455	0.060606	0.015152	0
MALY	0.385053	0.076821	0.096765	0.035033	0.015004	0
PACA	0.014848	0.002973	0.001647	0.004413	0.001074	0
RECA	0.014686	0.005591	0	0	0.001778	0
OV	0.036286	0.001305	0.010106	0	0.001092	0
BRCA	0.001019	0	0	0	0	0

Table S8. The OV cancer sample subtypes assigned by Subtype-WGME for the OV dataset, Related to Figure 5.

**donor_unique_id**	**Subtype**
OV-AU::AOCS-171	1
OV-US::f0c353fd-947c-41e2-b643-3ecc0d69796c	2
OV-AU::AOCS-117	2
OV-US::eeb9d147-608d-4692-8adf-2f601d23a8ff	3
OV-AU::AOCS-128	1
OV-AU::AOCS-134	2
OV-AU::AOCS-163	1
OV-AU::AOCS-086	1
OV-AU::AOCS-057	1
OV-AU::AOCS-080	1
OV-AU::AOCS-161	1
OV-US::e43d3769-e25f-4fb7-9080-ea26defaf094	2
OV-US::4eac2c98-86d2-4ee6-a1d3-157d013c78dc	3
OV-AU::AOCS-005	1
OV-AU::AOCS-065	1
OV-AU::AOCS-108	1
OV-AU::AOCS-119	2

OV-US::58d34254-4f5b-40a4-9e9f-7160062fb2a4	2
OV-AU::AOCS-170	1
OV-AU::AOCS-097	1
OV-US::9fb1ba57-2007-4477-b000-2d36f163efd2	2
OV-AU::AOCS-083	1
OV-AU::AOCS-105	1
OV-AU::AOCS-106	1
OV-AU::AOCS-153	1
OV-US::0f530b3e-5b6d-4892-9ebd-9138d76fdca7	3
OV-AU::AOCS-114	1
OV-AU::AOCS-061	1
OV-AU::AOCS-090	1
OV-AU::AOCS-115	1
OV-AU::AOCS-158	1
OV-US::5e18b17d-4626-4b6d-8ac6-e560cee0376c	3
OV-AU::AOCS-095	1
OV-AU::AOCS-058	1
OV-AU::AOCS-139	1
OV-AU::AOCS-004	1
OV-AU::AOCS-109	1
OV-AU::AOCS-111	1
OV-AU::AOCS-162	1
OV-AU::AOCS-034	1
OV-AU::AOCS-093	1
OV-AU::AOCS-084	1
OV-AU::AOCS-059	1
OV-AU::AOCS-165	1
OV-AU::AOCS-166	3
OV-AU::AOCS-104	1
OV-AU::AOCS-060	1
OV-AU::AOCS-138	2
OV-US::3c037acf-f453-4513-a6dd-129163ddde2a	2
OV-AU::AOCS-112	3
OV-AU::AOCS-001	1
OV-AU::AOCS-055	3
OV-AU::AOCS-159	1
OV-AU::AOCS-160	1
OV-AU::AOCS-096	1
OV-AU::AOCS-077	1
OV-AU::AOCS-091	1
OV-AU::AOCS-092	1
OV-AU::AOCS-079	1
OV-AU::AOCS-120	2

OV-AU::AOCS-164	1
OV-AU::AOCS-075	1
OV-AU::AOCS-085	1
OV-US::29949b65-2913-4c86-bbd7-b85c5df6f15e	1
OV-AU::AOCS-168	1
OV-AU::AOCS-113	1
OV-AU::AOCS-056	1
OV-AU::AOCS-141	2
OV-AU::AOCS-157	1
OV-US::700e91bb-d675-41b2-bbbd-935767c7b447	2
OV-AU::AOCS-150	2
OV-AU::AOCS-063	1
OV-AU::AOCS-094	3
OV-AU::AOCS-064	1
OV-US::30b8f4cd-9245-4496-a8a8-c3e59093bc0a	2
OV-AU::AOCS-142	2
OV-AU::AOCS-116	1
OV-AU::AOCS-167	2
OV-US::ee0a4a13-613e-4c5d-96c3-8083a013702d	3
OV-AU::AOCS-081	1
OV-AU::AOCS-107	1
OV-AU::AOCS-088	1
OV-AU::AOCS-169	1
OV-AU::AOCS-155	2
OV-AU::AOCS-078	1

Table S10. Biomarker numbers found by three algorithms, Related to STAR Methods.

Code	Randomforest	XGBoost	LightGBM
CLLE	4	2	1
ESAD	6	4	2
MALY	31	15	23
OV	31	8	19
PACA	49	29	19
PAEN	14	13	17
RECA	11	11	8
BRCA	49	35	50
Total	**195**	117	139
